# Comparative analyses identified species-specific functional roles in oral microbial genomes

**DOI:** 10.1080/20002297.2017.1325185

**Published:** 2017-06-26

**Authors:** Tsute Chen, Prasad Gajare, Ingar Olsen, Floyd E. Dewhirst

**Affiliations:** ^a^ Department of Microbiology, The Forsyth Institute, Cambridge, MA, USA; ^b^ Department of Oral Biology, University of Oslo, Oslo, Norway

## Abstract

The advent of next generation sequencing is producing more genomic sequences for various strains of many human oral microbial species and allows for insightful functional comparisons at both intra- and inter-species levels. This study performed *in-silico* functional comparisons for currently available genomic sequences of major species associated with periodontitis including *Aggregatibacter actinomycetemcomitans* (AA), *Porphyromonas gingivalis* (PG), *Treponema denticola* (TD), and *Tannerella forsythia* (TF), as well as several cariogenic and commensal streptococcal species. Complete or draft sequences were annotated with the RAST to infer structured functional subsystems for each genome. The subsystems profiles were clustered to groups of functions with similar patterns. Functional enrichment and depletion were evaluated based on hypergeometric distribution to identify subsystems that are unique or missing between two groups of genomes. Unique or missing metabolic pathways and biological functions were identified in different species. For example, components involved in flagellar motility were found only in the motile species TD, as expected, with few exceptions scattered in several streptococcal species, likely associated with chemotaxis. Transposable elements were only found in the two Bacteroidales species PG and TF, and half of the AA genomes. Genes involved in CRISPR were prevalent in most oral species. Furthermore, prophage related subsystems were also commonly found in most species except for PG and *Streptococcus mutans*, in which very few genomes contain prophage components. Comparisons between pathogenic (P) and nonpathogenic (NP) genomes also identified genes potentially important for virulence. Two such comparisons were performed between AA (P) and several *A. aphrophilus* (NP) strains, and between *S. mutans* + *S. sobrinus* (P) and other oral streptococcal species (NP). This comparative genomics approach can be readily used to identify functions unique to or prevalent in a group of genomes. It can work with annotation systems using structured functional terms, such as RAST, KEGG and Gene Ontology, and obtain comprehensive functional insights into microbial genomics.

